# Machine Perfusion for Extended Criteria Donor Livers: What Challenges Remain?

**DOI:** 10.3390/jcm11175218

**Published:** 2022-09-03

**Authors:** Jeannette Widmer, Janina Eden, Mauricio Flores Carvalho, Philipp Dutkowski, Andrea Schlegel

**Affiliations:** 1 Department of Surgery and Transplantation, Swiss HPB Centre, University Hospital Zurich, 8091 Zürich, Switzerland; 2 Hepatobiliary Unit, Department of Clinical and Experimental Medicine, University of Florence, AOU Careggi, 50139 Florence, Italy; 3 Fondazione IRCCS Ca’ Granda, Ospedale Maggiore Policlinico, Centre of Preclinical Research, 20122 Milan, Italy

**Keywords:** extended criteria donors, donation after circulatory death, machine perfusion, mitochondria

## Abstract

Based on the renaissance of dynamic preservation techniques, extended criteria donor (ECD) livers reclaimed a valuable eligibility in the transplantable organ pool. Being more vulnerable to ischemia, ECD livers carry an increased risk of early allograft dysfunction, primary non-function and biliary complications and, hence, unveiled the limitations of static cold storage (SCS). There is growing evidence that dynamic preservation techniques—dissimilar to SCS—mitigate reperfusion injury by reconditioning organs prior transplantation and therefore represent a useful platform to assess viability. Yet, a debate is ongoing about the advantages and disadvantages of different perfusion strategies and their best possible applications for specific categories of marginal livers, including organs from donors after circulatory death (DCD) and brain death (DBD) with extended criteria, split livers and steatotic grafts. This review critically discusses the current clinical spectrum of livers from ECD donors together with the various challenges and posttransplant outcomes in the context of standard cold storage preservation. Based on this, the potential role of machine perfusion techniques is highlighted next. Finally, future perspectives focusing on how to achieve higher utilization rates of the available donor pool are highlighted.

## 1. Introduction

With the first successful transplantation of a whole liver, donated after circulatory death (DCD), Starzl et al. launched the era of liver transplantation (LT) in 1963 [1,2]. Based on regulations and guidelines at that time, the use of this type of liver from a so-called extended criteria donor (ECD), as we would classify it today, was easily accepted. Additionally, many risk factors that contribute to impaired outcomes were already described. In order to minimize the impact of donor warm ischemia time (DWIT), the organs were immediately cooled by a flush of infusion through the portal and arterial system or by the use of a simple, extracorporeal hypothermic perfusion circuit [3]. In the seventies, a growing scientific understanding led to changes in the regulatory framework towards the use of organs donated after brain death (DBD) [4,5]. The general evolvement of surgical and medical treatment concepts, together with the introduction of immunosuppressive regimens, resulted in an overall success story of solid organ transplantation.

**Figure 1:**
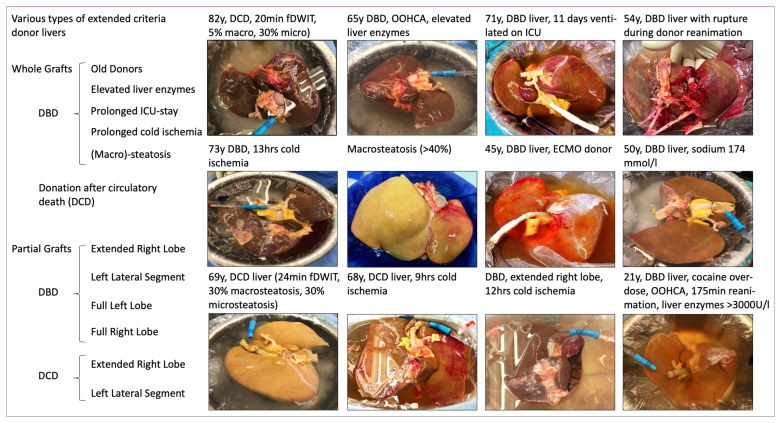
Overview of different types of donor livers classified “extended” based on the functional risk. The livers in Figure 1 were procured from and assessed in the authors’ transplant centers. All livers underwent hypothermic oxygenated perfusion (HOPE) for evaluation, and decision making was based on mitochondrial function and injury during HOPE. DBD: donation after brain death; DCD: donation after circulatory death; ECMO: extracorporeal membrane oxygenation; fDWIT: functional donor warm ischemia time; ICU: intensive care unit; HOPE: hypothermic oxygenated perfusion; micro and macro: micro- and macrosteatosis; OOHCA: out-of-hospital cardiac arrest.

One direct consequence appears with an ever-increasing number of candidates listed for transplantation, along with a growing acceptance of new indications, including primary and secondary liver tumors. To serve this large pool of recipients, clinicians accept livers from donors with a completely different risk profile today compared to previous decades. Donor livers with increased risk are classified as ECD today. Although a few “classic” risk factors, such as donor age or DCD, are considered by many, this group of livers is the most heterogenous cohort identified (Figure 1).

Of note, regulations and ethical considerations in countries, regions, centers and even among individual surgeons impact the definition of organ marginality. The best example appears with donor age. While DCD livers with an age beyond 50 y are considered as extended risk in the United States (US), such grafts are routinely transplanted with standard cold storage (SCS) in experienced centers in some European countries [6,7]. Similar features are seen with any other donor and recipient risk factor, pointing towards the high impact of personal and center experiences [8]. Another contributing factor is the increasing understanding of underlying mechanisms of ischemia–reperfusion injury (IRI) and strategies on how to reduce the impact of this inflammatory cascade on outcomes after LT.

With increasing experience, the criteria of acceptable donor quality were stretched and balanced with the recipient risk factors in several prediction models [9,10,11,12]. Previously discarded organs are frequently classified as “standard” or even “good quality grafts” today. Based on an increasing number of publications where the authors describe various parameters with an impact on the outcomes, the term ECD covers a pool of grafts from donors with countless risk profiles and specifications that lack a uniform terminology and guidelines [13,14,15,16].

The underlying mechanisms of injury described after the reperfusion of cold-stored ECD livers point to a high relevance of the mitochondrial metabolism, which is directly linked to the overall metabolic situation in the donor [17,18,19]. The role of the established simple and cost-effective SCS preservation is therefore increasingly challenged. Novel organ perfusion strategies are being nominated to improve the current concepts with a focus on better organ function, reduced complications and improved graft and patient survival after LT [17,20].

Such concepts of dynamic organ preservation provide the additional opportunity to test organ viability before implantation to safely increase the use of ECD organs [21,22].

This review provides an overview on the various types of ECD livers and related classifications used today. Mitochondria are another focus, and their role as key instigators of the inflammatory IRI cascade and as targets for dynamic preservation strategies is highlighted. Finally, the clinical outcomes achieved with different perfusion strategies are described and challenged among each other together with a future perspective and remaining challenges in this interesting field.

## 2. Types of Extended Criteria Donor Livers and Associated Risk Factors

### 2.1. A General Overview

While regulations and center policies certainly impact decision making, the classification of a donor liver, as ECD is frequently based on the surgeon’s experience or “gut feeling” [8,23]. Despite the recognition of standard risk factors with well-defined cut-offs, donor livers exceeding a threshold of one parameter would not automatically be discarded by every transplant surgeon in the same center or beyond [8]. Some criteria represent a higher risk, while others indicate a real threshold where most clinicians would decline an organ. The caseload and the risk a center is willing to accept impact these decisions further. With the increasing need for a better utilization of the donor pool, the standard graft today would have been classified as an ECD type a decade ago (Table 1).

The heterogenous group of ECD livers covers two main groups: first, livers with the risk for poor graft function, and second, livers from donors with a specific disease with the potential for transmission to the recipient. With the increasing experience with donor infections and tumors, fairly clear guidelines on what to accept are available and widely respected [24,25,26,27]. In contrast, specific criteria to describe donor parameters with the potential to trigger high levels of IRI with more complications and impaired graft and patient survival appear less well-defined.

The Eurotransplant definition includes the following criteria for ECDs: donor age > 65 years; intensive care unit (ICU) stay of >7 days with ventilation; body mass index (BMI) > 30 kg/m^2^; liver steatosis of >40% and elevated serum sodium, transaminases and bilirubin [13,27]. There are, however, numerous reports of successful transplantations with livers exceeding these cut-offs, and from the recent literature, we can see a trend towards benchmark outcomes for ECD allografts [28]. Additionally, various reports consider slightly different thresholds for specific donor risk factors, particularly for donor age and cold ischemia time (CIT). Goff et al. recently suggested to consider the 90th percentile of the donor risk index (DRI) to nominate livers as ECD organs [29].

The criteria to routinely accept whole grafts for two recipients appear generally stricter. This is due to, first, the split procedure, which adds further risk with the release of more proinflammatory molecules, an expected prolonged CIT based on increased liver travel time and implantation using smaller vessels. Split liver transplantation is therefore nominated by many as an extended risk.

While most authors focus on the parameters, which can be objectively quantified with a scale, reports from livers with severe procurement injuries or lacerations from a donor reanimation or trauma are scarce. Such livers may well transmit a very high risk for complications and are frequently discarded by surgeons, whose experiences are of utmost importance [8,30].

The following sections describe the roles of various types of ECD livers in IRI and posttransplant complications, along with suggested thresholds for acceptance.

### 2.2. Donation after Circulatory Death (DCD) Livers

With the development of the Harvard criteria for brain death in 1968 and the acceptance of the declaration of death according to the neurologic criteria as a legal entity, most transplant centers exclusively implanted DBD allografts [4]. In the early nineties, the utilization of DCD organs regained interest due to an increasing organ scarcity [31]. The transplantation of DCD allografts continuously grew, ranging between 1.7% and 40% of all deceased donors [32]. In Europe, 18 countries have a more or less active DCD liver transplant program [33]. Based on the overall increasing experience with a better understanding of related donor risk parameters worldwide, a recent benchmark analysis demonstrated similar results after DCD and DBD LT [34,35]. The key concern remains an early non- or dysfunction and the development of biliary complications, which are directly linked to the higher cumulative donor risk with warm ischemia [35,36]. Prolonged DWIT is associated with higher IRI levels and more complications. Livers from donors with metabolically inferiority seem unable to recover from the reperfusion hit, and the subsequent ongoing inflammation leads to an early fibrosis and related complications, including ischemic cholangiopathy (IC) [17,36].

Although DWIT is the most prominent risk factor in DCD transplantation, uniform definitions and widely accepted thresholds are still lacking, and each country, center or even surgeon follows slightly different guidelines.

Three main definitions for DWIT are established and, for all, applies the same rule: “The shorter, the better”. The total DWIT (time from donor treatment withdrawal to cold flush) includes the agonal phase (from donor treatment withdrawal to circulatory death) and the asystolic DWIT (circulatory death to cold flush). The best timepoint when to start the functional DWIT calculations is under debate and depends on the recognition of both donor hypotension and hypoxia after treatment withdrawal. A systolic or mean arterial blood pressure of <50 mmHg is frequently considered as the entrance point for functional DWIT [11,37,38]. Levels of hypoxia are, however, increasingly recognized when falling below a pO2 of 70 or 80 kPa [37]. Such inhomogeneous guidelines are the one remaining challenge to reliably compare the results of current studies, where the range of specific posttransplant complications appears equally enlarged [35,39]. During a recent consensus conference in 2020, the committee suggested to measure the functional DWIT according to the Spanish guidelines, starting when hypotension with systolic blood pressure of <60 mmHg or saturation below 80 kPa was seen in the donor [40].

With the introduction of such definitions, new studies are required to compare outcomes according to this newly key donor risk factor. A functional DWIT duration, which was previously based on systolic blood pressure below 50 mmHg and saturation below 70 kPa, will appear longer with the new definition with a presumed higher risk, because the injury is captured earlier. Donors will more frequently cross the upper limit to be accepted for LT with a functional DWIT beyond 30 min, the current cut-off in Spain, France and other countries with a DCD program [32]. With an expected higher liver discard rate in the context of SCS preservation, the guidelines will require adaptations. An “ideal DCD donor” proceeds with a functional DWIT of 20 min or less [11]. The wide range of country-specific “donor stand-off periods” between 5 and 30 min impacts such timings further [32]. Italy is a leading country for DCD LT. Centers routinely apply different dynamic perfusion approaches based on their 20-min stand-off period, and the entire DCD liver population is classified as extended with a median functional DWIT of >40 min in recent series and clearly beyond any suggested threshold seen in other countries, except for Switzerland (Table 1) [32,41,42].

In a recent benchmark study, DCD livers with >15 min asystolic and >30 min total DWIT were classified as high risk, and the recipients demonstrated significantly higher rates of IC-related and overall graft loss [35]. Such DWIT timings were previously paralleled by many, and the link between a prolonged asystolic DWIT of >15 min and later IC was described [36].

### 2.3. Donor Age

With the limited capability to recover from a metabolic insult, elderly grafts are frequently discussed to suffer elevated IRI levels with more complications after LT, particularly in combination with prolonged warm or cold ischemia [43]. Even healthy aged livers demonstrate various morphological transformations, including a higher parenchymal stiffness, arteriolar walls thickening with a lower perfusion quality and additional changes in the immune system [44,45].

Aging further decreases the capability of the liver to regenerate [46]. On the cellular level, mitochondria are increasingly recognized as a key target of age-related susceptibility to injury. During reperfusion, higher levels of oxidative stress were observed in older organs, followed by an impaired restoration of the metabolic function during reperfusion [47,48]. Donor age is therefore always recognized as an important risk factor in solid organ transplantation, and most studies categorically advise against the use of livers from donors beyond 60 y, 65 y, 70 y or 80 y [49,50,51].

While most guidelines, including those provided by Eurotransplant and the EASL list and a donor age > 65 y as a primary ECD criterion, such grafts are routinely used in different countries with an otherwise long waiting time for a graft [50,52,53]. While, in the 90 ties where an American study found significantly higher primary non- and delayed graft function rates (PNF and DGF) with elevated donor age > 50 y, good results were reported more recently, with an overall improved medical and surgical donor and recipient management [54]. A study from a large transplant center in Italy has demonstrated equally low complications and good graft survival with the use of octogenarian livers [50]. Advanced donor age is therefore increasingly considered as the “standard risk” today. A few reports show the safe utilization of livers from donors > 90 years, provided other risk factors are controlled [55,56]. Although the outcomes are equally good, a higher risk for biliary complications was described recently in a meta-analysis [57].

Next, higher incidences of hepatic artery thrombosis (HAT) were previously reported after LT from older donors [58]. The experiences of the donor and recipient surgeon, together with the quality of the vascular liver supply, are crucial and impact the outcomes.

Based on the increasing experiences with elderly grafts, donor age is routinely listed as a contributing risk factor in many scoring systems [11,12,59,60]. Although a donor age > 65 years alone is rarely considered as the key criterion to decline a liver offer, higher complication rates such as PNFs were described when other risk factors, including small grafts, steatosis, long CIT or DCD, were evident [61,62,63,64].

Several studies showed a linear risk increase for graft failure of 12% per additional donor age decade [51,65]. Various thresholds were nominated by >40 y or >60 y, with clearly more complications beyond [12]. Though livers from advanced aged donors are preferably transplanted into older candidates (“old for old”), there is evidence that younger recipients may have the ability to “rejuvenate” an “old” liver [66]. The final acceptance of donor livers within a certain age depends on additional donor risk factors but also on the recipient conditions and the expected time a candidate might be able to wait for a younger graft.

Similar features were seen with the combination of donor age and warm ischemia in DCD transplants. In 2009, the ASTS guidelines were published to advise for caution when DCD livers > 50 years were offered. In contrast, good results for LT with elderly DCD donors were also shown [6,67]. Two studies demonstrated similar outcomes after the transplantation of DCD livers from donors beyond 60 y and 70 y with a well-controlled overall risk.

### 2.4. Cold Ischemia

From donor cardiac arrest to reperfusion of the organ in the recipient, different types of ischemia affect the allograft. The frequently discussed CIT starts during procurement when the organ is coldly perfused and ends when the implantation starts. It is a modifiable factor depending on organ allocation policies, donor/recipient management and the preservation technique. It is highly associated with graft and patient survival that declines in a linear fashion as CIT increases. With each additional hour of CIT, the risk of a posttransplant graft was found to increase by 3.4% [61]. Already, in 1992, prolonged CIT of >12 h was listed as a risk factor for impaired graft function and patient survival. A retrospective study set the optimal cut-off for CIT at 8 h [62]. Another aspect was shown by the group from Heidelberg. Recipients with HCV cirrhosis tolerated an increase of CIT less well compared to recipients with cirrhosis due to alcoholic liver disease [61]. Based on the modifiable feature of CIT, the overall aim is to keep this parameter as short as possible (ideally below 6 or even 4 h) or to avoid additional risk factors in combination with a longer CIT.

For DCD livers, a recent consensus conference suggested to accept such grafts with a CIT of up to 8 h and to consider the role of additional risk factors when the CIT may be prolonged [68]. The reason behind this is a higher IC rate seen with prolonged CIT, which is also linked to a longer hospital stay and higher PNF rates in DCD recipients. Each additional hour increases the risk by 11% in DCD allografts [69].

### 2.5. Steatosis

Liver steatosis is a growing challenge not only in deceased donors but also for living donations [70,71,72]. Elevated liver steatosis leads to sinusoidal narrowing and subsequent impaired tissue perfusion with hypoxia already in the donor, particularly with a prolonged treatment in the ICU [73]. In combination with an extended cold or even warm ischemia, this leads to a high risk for severe IRI and complications after transplantation, including IC [71,74].

Donor liver biopsies are considered helpful to differentiate between micro- and macrosteatosis, further specified into large and small droplets [75]. Both histological types of steatosis are divided into mild (>30%), moderate (30–60%) and severe (>60%) [76]. The small amount of tissue obtained for biopsies appears to not represent a reliable estimate of the overall liver fat content. Pathologists aim to provide a percentage of affected hepatocytes with rather low accuracy [77]. Surgical experience with macroscopic liver assessments therefore still plays an important role in supporting decision making, while machine perfusion (MP) techniques are explored to identify more reliable markers for viability assessments. New techniques to quantify the fat in the entire organ are currently being explored, including MRI and spectroscopy [78,79]. A large study with 20,000 transplants from the US and Europe demonstrated the safe use of microsteatotic grafts for recipients of all risk levels, according to the balance of risk (BAR) score [15]. This study was, however, based on large data registries, which have inherent challenges with the data accuracy and a fairly high number of missing cases.

Croome et al. recently explored the impact of >30% microsteatosis and showed a higher rate of postreperfusion syndrome, early allograft dysfunction (EAD) and dialysis after LT compared to non-steatotic livers [80]. The overall graft and patient survival were, however, similar. Microsteatotic livers are therefore routinely allocated to candidates capable of handling the initial inflammatory IRI hit, an “unwritten rule” respected by many for all sorts of ECD livers [80]. Such results confirm clinically the previously described process of defatting in the recipient within a few weeks after transplantation [81].

In contrast, the number of transplantations described in the literature with the use of severely macrosteatotic livers is limited [82]. A recent study demonstrated an 8.3% risk for cardiac arrest after the transplantation of macrosteatotic livers (>30–60%) compared to 1% and 0%, seen with the use of mild and non-steatotic grafts [83]. Such results parallel a previous work, where the authors recommended the selective use of moderately macrosteatotic livers for rather “fit” recipients with bar scores of <9 points. In contrast, livers with mild steatosis were recommended for the entire recipient population up to an overall risk of 18 bar score points [15]. The underlying mechanisms are linked to mitochondrial dysfunction and the release of reactive oxygen species (ROS) and other proinflammatory molecules within the first minutes after reperfusion, which were identified with much higher levels in ECD livers, including steatosis [72,84].

Such mechanistical features are even more prominent when steatosis is combined with DWIT, where specific data are, however, scarce [71]. Croome et al. found higher rates of cardiac arrest, PNF, EAD and acute kidney injury with moderately macrosteatotic DCD grafts (>30%) compared to the transplantation of DCD livers with no steatosis [71]. Of note, the results should also be analyzed in the context of the rather short DWIT seen in this young liver cohort from the US, which could be the reason for rather good outcomes with a macrosteatosis of up to 30% [35,68,71].

Although the BMI is an imperfect surrogate marker for liver steatosis, donors are frequently declined based on elevated values. While a strict policy is frequently lacking [85], higher rates of biliary and renal complications and impaired graft survivals are described based on donor BMI of >25 kg/m^2^ in DCD livers [67,86,87,88,89]. The threshold of >30 or even >35 kg/m^2^ is, however, more often recognized by many [11,68,71] and further supported by the new ILTS consensus guidelines [68]. Similar categorizations were suggested by Eurotransplant and the EASL guidelines for all ECD grafts (Table 1) [13,27].

The use of both donors with high BMI and/or livers with steatosis should always be based on the overall donor and recipient risk and in the context of the center’s experience [8]. The time required for the procurement and the quality of liver and bile duct flush, which are both based on the surgeon’s experience, further impact the outcomes [68,90]. Time pressure may also lead to a higher number of injuries to the organ or vascular structures [91,92]. Equally, such risky grafts should be matched individually to appropriate recipients [8,71]. Dynamic organ preservation techniques will change this landscape in the future.

### 2.6. The Role of Other Donor Risk Factors

Further risk is transmitted with the cause of donor death or elevated enzymes, prolonged ICU stay and the need for specific treatments, including extracorporeal membrane oxygenation (ECMO). Additional risk is transmitted through elevated donor sodium levels and an overall suboptimal donor management, seen in many countries worldwide. Elderly donors with a vascular event, including intracranial hemorrhage, are kept rather shortly in the ICU until donation and are considered good donors.

The British Transplantation Society (BTS) classifies DCD donors with an ICU stay of ≤5 days as low risk [38], and in the Eurotransplant region, donors with >7 days ICU stay with ventilation are considered marginal [13,27,93].

In contrast to the older population, younger donors are more frequently presented with a prolonged ICU stay after severe trauma or sudden out-of-hospital cardiac arrest (OOHCA) with relevant downtime and organ hypoxia before donation, resulting in irreversible hypoxic brain injury. Though the utilization of donors after OOHCA is routine today, the lack of uniform guidelines remains [94]. A heterogenous group of donor risk factors is considered a surrogate to predict later complications after LT. Such parameters include the required amount of medical cardiovascular support, the duration of the previous downtime and the levels of donor liver enzymes and function [95]. The Eurotransplant guidelines suggest the classification of livers as extended when serum transaminases cross the normal values greater than three times (Table 1) [13,27].

A previous downtime with organ ischemia and subsequent reperfusion during donor reanimation is also considered as protective, because similar to ischemic preconditioning, such events may prime mammalian tissues for later ischemia with the early upregulation of defense mechanisms [96]. The data and thresholds are, however, scarce, and most surgeons accept such organs, depending on the clear downtrend of transaminases and maintained liver function [95,97,98]. Based on this, some centers would use marginal livers with initial enzymes beyond 1000 U/L and also DCD grafts with elevated values of up to 300 U/L. These scenarios may also point to previous donor drug abuse.

Donor trauma and reanimation could also affect the major liver structures with parenchymal and vascular injuries. The surgical exploration of the organs is strongly recommended to assess if livers are transplantable, considering the surgical repair of injured structures (Figure 1) [8].

Additional risk may be conveyed through past medical donor history, which may have a negative impact on arterial vessels, the subsequent donor cannulation and may appear with suboptimal liver perfusion before and during donation. Such features may be related to donor arteriosclerosis, arterial hypertension, cardiomyopathy, right heart failure with the need for cardiac-assist devices or diabetes [99,100]. Due to the remaining difficulties in capturing the real impact of such parameters, many colleagues are reluctant to accept and even surgically evaluate these donor livers in the context of static cold storage preservation [99]. The rather strict recommendations when to accept split livers include, for example, a threshold for maximal cardiovascular support with 8 μg norepinephrine/kg/min or 15 μg dopamine/kg/min. Other guidelines suggest a cut-off for an acceptable dopamine donor dosage between 6 and 10 μg/kg/min [95].

### 2.7. Partial Grafts from Deceased Donors

Split liver transplantation, introduced in the eighties, appears as the key to reduce waiting list mortality, together with living-related liver transplants. One deceased donor liver is shared between two recipients, and the split procedure can be performed in- or ex situ, with inherent benefits to both approaches. The smaller left lateral segment is often used for children and the larger extended right lobe for an adult or adolescent candidate [101]. The thermal liver transection does, however, bear a higher risk for later post-reperfusion inflammation and related complications, particularly in combination with riskier vascular reconstructions in both pediatric and adult recipients. The liver has to handle the combined insult of reperfusion and is expected to recover its function and grow at the same time. More posttransplant complications were therefore described and led to strict donor selection criteria and recipient matching [102]. Although each European country has developed its own split policy, most risk factors in accepting a donor for splitting are commonly applied and include: a maximum donor age of 40–50 years, stable hemodynamic parameters, a donor ICU stay of <5 days, a sodium level below 160 mmol/L and a liver without steatosis. Organs accepted for splitting come from donors with liver enzymes of a maximal fivefold of the normal values [102].

A suitable vascular anatomy for later reconstruction and age/weight-related donor recipient size matching are routinely considered [102]. A recent analysis from the European Liver Transplant Registry found an overall 5-year graft survival of 72.9% and highlighted the following risk factors for 3-month graft failure: a donor age of >50 years, a CIT with a hazard ratio of 1.07 per additional hour, an urgent recipient status and a recipient body weight of ≤6 kg [102]. Respecting recently suggested guidelines, a donor age of up to 50 y is considered acceptable, provided the lack of other relevant risk factors. Children with a weight of less than 6 kg and those listed for an urgent transplantation should receive a split graft with a maximal CIT of 6 h, given that the graft and recipient size are matched [102].

Considering the current logistic challenges and the frequent traveling of grafts from the donor to the split and recipient center, a CIT of 6 h is a significantly limiting factor. The better understanding of risk factor boundaries, along with increasing experience (also with logistics) and the introduction of a mandatory split policy, as, for example, in Italy, can increase the utilization. DCD livers are rarely considered for splitting due to an overall too-high risk. More recently, MP techniques are used for these organs during and also after the split procedure, which will be presented in more detail below [101,103].

**Table 1:** Current ECD criteria focusing on the functional risk.

Parameter	ECD Categories: Eurotransplant/EASL [13]	Criteria Specification	ILTS Consensus Criteria 2020: Controlled DCD [68]	Criteria Specification	Split Criteria (ELTR Criteria) [102]	Criteria Specification
Functional donor risk factors	Donor age > 65 y	Donor age >70 y [29], >80 y [50], >90 y [55,56]	Donor age ≤ 60 y	Donor age > 60, >70	Donor age ≤ 40 y (10–40 y)	≤50 y, variable in different counties [102], 18–40 y [29,101]
	Donor BMI > 30 kg/m^2^		Donor BMI ≤ 30 kg/m^2^	Explore BMI > 30 kg/m^2^ donor with experienced donor surgery team [68]	Donor BMI ≤ 30 kg/m^2^	Donor BMI < 28 kg/m^2^ [29,101]
	Donor ALT > 105 U/L, Donor AST > 90 U/L (“>3-times normal”)	Higher transaminases (>1000 U/L) when down-trend is confirmed and liver maintained [95,97,98]		Downwards-trend maintained function, individual decision [68]	Donor AST and ALT <5-fold normal values	Donor AST and ALT <3-fold normal values [101]
	Donor serum sodium > 165 mmol/L	Donor serum sodium > 180 mmol/L			Donor serum sodium > 160 mmol/L	Selective use of donors with sodium > 160 mmol/L
	Donor serum bilirubin > 3 mg/dL					
	Donor ICU stay with ventilation > 7 days			BTS guidelines suggest ≤ 5 days donor ICU stay [38]	Donor ICU stay with ventilation ≤ 5 days	
	Liver steatosis > 40%	Macrosteatosis > 30% [29]	Macrosteatosis ≤ 30%		No relevant steatosis	
	DCD donor [13]				Only DBD	DCD livers very selectively
	Cold ischemia time > 14 h [13] *	>10.5 h [13] *	Cold ischemic time ≤ 8 h	Keep as short as possible	Cold ischemia time ≤ 8 h (>8 h increase risk for ERL [101])	6 h for urgent and <6 kg recipient weight [102]
		Donor cardiac arrest [13] National share [29] 90 th percentile of DRI [29]				Donor body weight > 40 kg (Italy) > 50 kg BTS, [101]
						Single vasopressor [29,101]
Recipent risk factors			Lab MELD ≤ 25 points	Selective use in recipients with lab MELD > 25 points [68]		Age, weight, status, availability of LDLT graft, waiting time are among additional factors of impact
			Selective use in NASH recipients			
			No known complex PVT, selective use for re-TPL or acute liver failure or combined liver-kidney transplant	No specific recipient age cut-off, high or low, selective use in pediatric candidates [68]		
Donor risk factors, not relevant for graft function	Positive hepatitis serology [13]		Consider donor infections and malignancy according to WHO/EASL guidelines			
	History of extrahepatic malignancy [13]					

Donors/Livers beyond the ECD Eurotransplant/EASL criteria are considered as extended, and the guidelines did initially target extended DBD grafts, while DCDs and a certain cold ischemia time were also considered risk factors but not listed in the original criteria. **Criteria specifications** describe additional modifications of established criteria by different authors, and such livers are routinely considered when other risk factors are well-controlled or machine perfusion is available. Several case series exist with the use of old grafts beyond a donor age of 80 or even 90 y. * Particularly in combination with other donor risk factors, a cold ischemia time of >14 h is often considered a high risk, and most centers would probably set the cut-off at lower values and use machine perfusion. Reference [13] describes the Eurotransplant/EASL criteria and additional in-house criteria in Graz. The ILTS consensus meeting in 2020 defined the criteria for controlled DCD livers that should be routinely used in the context of standard cold storage, recipient risk, center policy and experience. DCD livers beyond the suggested thresholds should be used in the context of machine perfusion and with caution and considering other donor risk factors (including donor age, BMI, functional DWIT, macrovesicular steatosis, hospital stay, trends in donor liver function tests and donor hepatectomy time) and recipient risk factors. Split policies differ in each country. The ELTR criteria for the acceptance of a liver to split: donor age can be safely extended to 50 y. Younger donors < 10 y convey additional risks for graft loss. Individual decisions beyond suggested cut-offs. ALT: alanine aminotransferase; AST: aspartate aminotransferase; BMI: body mass index; BTS: British transplantation society; DCD: donation after circulatory death; DRI: donor risk index; EASL: European Association for the Study of the Liver; ECD: extended criteria donor; ELTR: European liver transplant registry; ERL: extended right lobe; ILTS: International Liver Transplant Society; MELD: model of end stage liver disease; PVT: portal vein thrombosis; TPL: transplantation; WHO: World Health Organization. The numbers in brackets correspond to the references.

## 3. The Role of Recipient Risk Factors

Donors and grafts are always accepted in the context of the overall recipient status and risk. The entire spectrum, including recipient age, BMI, lab MELD and the medical history, is considered together with the technical risk parameters, such as advanced portal vein thrombosis or a candidate listed for retransplantation, are of particular importance [68]. Such factors are well-described in the literature as contributors of impaired outcomes already with optimal grafts, and a recent DCD liver transplant consensus conference further pronounced their impact when combined with the additional donor risk [68,104,105]. With improved medical and anesthesiologic recipient management, the overall number of liver transplant recipients beyond 65 y of age has increased worldwide [68]. Older candidates are, however, known to more frequently drop out from the waiting list due to their comorbidities. This cohort is otherwise known for a higher waiting list mortality despite a lower lab MELD [68]. Inferior 5-year survival rates were described in candidates between 60 and 70 years, which are also linked with donor age (e.g., >60 years) and the cumulative donor risk [105]. One reason behind this is the role of comorbidities, particularly those of cardiovascular and renal origin.

A team from Leeds found an additional increasing number of complications with an elevated recipient BMI of >24.9 kg/m^2^. Values above >29.9 kg/m^2^ were linked with significantly more infections and a prolonged hospital stay when compared to recipients with a normal body weight [68,105]. The use of DCD livers led to more renal complications after LT in the cohort with a recipient BMI of >30 kg/m^2^ [89].

The recipient disease severity is another contributor, and patients with a lab MELD of >30 points are known for their higher risk of intra- and postoperative complications with prolonged ICU and hospital stays. In combination with advanced age, an elevated recipient lab MELD of >25 points was linked to higher early posttransplant mortality rates, when, for example, combined with DCD grafts or an otherwise elevated donor risk and in the context of SCS [12,68].

Of additional impact is the underlying recipient liver disease, with a general reluctance to allocate ECD livers to candidates with non-alcoholic steatohepatitis (NASH), a recipient cohort known for their relevant comorbidities being prone to cardiovascular complications [106].

## 4. Mechanisms of Ischemia-Reperfusion Injury with the Use of ECD Livers

During ischemia, cellular hypoxia leads to an invisible instigation of the rather complex IRI cascade. The key players here are mitochondria—the powerhouse and energy provider in all sorts of mammalian cells. The most exclusive mitochondrial function is cellular respiration, with the final product crucial to the survival of all cells: adenosine triphosphate (ATP). A cascade of enzymatic reactions of the tricarboxylic acid (TCA) cycle generates reducing metabolites to transfer electrons across the mitochondrial respiratory chain, where electrons are funneled through complex proteins I–IV to generate potential across the inner mitochondrial membrane, which is required to produce ATP. This process is strictly oxygen-dependent [107,108].

**Figure 2:**
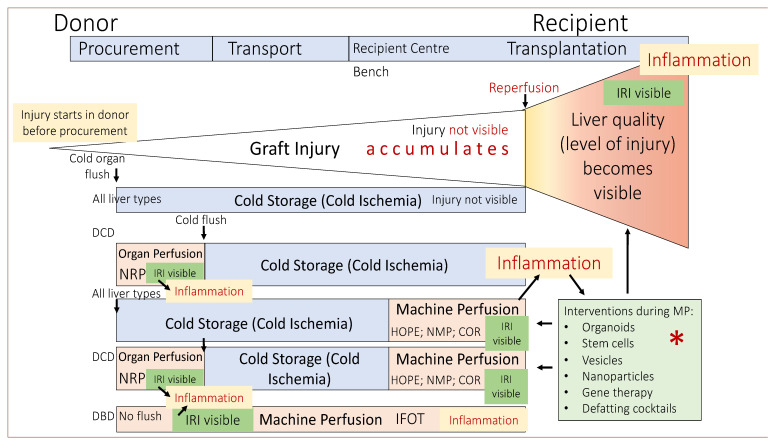
Pathway of injury during organ donation, preservation and after implantation. The reintroduction of oxygen causes IRI immediately and within the first few seconds. Downstream inflammation is only a consequence that unveils the real organ quality. The temperature has a significant effect on the level of IRI, with limited inflammation during cold reoxygenation compared to the warm conditions. The donor and graft quality and the capability to sustain function and recover after brain death and donor treatment in the ICU are the key factors for later organ function and recipient complications in the context of cold storage preservation. * Novel interventions aim to reduce the already established IRI inflammation, instead of focusing on the prevention of this cascade prior to rewarming. DBD: donation after brain death; DCD: donation after circulatory death; HOPE: hypothermic oxygenated perfusion; IFOT: ischemia-free organ transplantation; COR: controlled oxygenated rewarming; NMP: normothermic machine perfusion; NRP: normothermic regional perfusion. Figure done supported by biorender.com (assessed on 21 July 2022).

One important enzyme, participating in both the TCA-cycle and the electron transport chain, is succinate dehydrogenase (SDH). During ischemia and with the lack of oxygen, cellular respiration switches from aerobic to anaerobic. Molecules, including NADH and succinate, and other precursors of the TCA cycle accumulate, and the cells suffer a lack of energy with steadily decreasing ATP with prolonged ischemia and due to nonfunctioning cellular respiration [109,110]. In addition, electrolyte transporters in the cell membrane are in the off function, with subsequent cellular swelling based on a nonphysiological sodium–potassium shift with the accumulation of intracellular sodium [90,91]. One direct consequence is the increased cellular calcium levels and the activation of calcium-sensitive enzymes. Such complex mechanisms appear invisible, and despite increasing experience throughout the clinical work, transplant surgeons cannot predict which organ will suffer severe IRI and should decline them just by the macroscopic assessment. In the second step, with the reintroduction of oxygen (=reperfusion), the level of IRI injury becomes suddenly visible (Figure 2).

The functions of the respiratory chain are reactivated, and the electron flow is immediately restored. As a result, and with the proper function of a major proportion of the mitochondria in a cell, the levels of ATP increase and may fuel the augmenting metabolic demand at higher temperatures [107,108,110]. Previously accumulated succinate is oxidized by complex-II (SDH), thereby reactivating the process of proton-pumping across the inner mitochondrial membrane and leading to a reverse electron transfer (RET). The subsequent production and release of ROS from complex-I is the direct consequence [16,17,92].

The higher the injury (e.g., ECD grafts) with high succinate levels, the more ROS molecules are released from complex-I and pass through the inner mitochondrial membrane to cause injury to other cellular components, initiating a downstream cascade of tissue injury and inflammation. The first wave of IRI is the hyperacute injury, where the ROS and DAMPs (damage-associated molecular patterns) are released from all cell types and contribute to the acute inflammation of the overall tissue, characterized by type modifications of macrophages and their additional contribution to the cascade. This scenario is followed by the chronic phase, where circulating recipient immune cells, e.g., monocytes, are recruited and infiltrate the newly implanted organ to further enhance the injury.

Various cytokines and chemokines are released and recirculate to enhance and endure the ongoing inflammation. Once prolonged, ongoing inflammation leads to the development of tissue fibrosis and stiffness with clinical signs of example biliary complications, such as ischemic cholangiopathy.

The impaired capability of cells to regenerate, e.g., hepatocytes and cholangiocytes further aggravates this. The extent of this IRI tissue injury is ultimately associated with posttransplant complications, including early allograft dys- or nonfunction. The level of IRI is directly linked with the donor quality, and every manipulation and additional injury between the donor and recipient contributes further. Key interventions should therefore target the mitochondria as the main initiators of this injury and reduce or ideally prevent the accumulation of succinate with the subsequent reduction of ROS release. Despite the general awareness of such mechanisms for many decades, novel treatments are being increasingly developed to block downstream inflammation instead of preventing such an injury in the mitochondria right at the root of IRI. Cytosorb filters are one example of an expensive add-on during MP with a still not well-described effect but high contributions to the cost [111,112].

## 5. The Impact of Dynamic Organ Perfusion Strategies

Based on the above-described mechanisms, a detailed plan was recently provided on how to prevent significant IRI features. Two main options to prevent succinate accumulation and engery loss were evaluated: first, in the donor, or early during the donation. The second option implies a continuous metabolism of succinate together with ATP-reloading, a mechanism requiring the reintroduction of oxygen (reperfusion), which should, however, be achieved without the “price” of ROS release [107].

Currently explored dynamic preservation concepts span the same goal to provide oxygen to preinjured ischemic tissues and to avoid additional injury, e.g., further succinate accumulation. Such concepts are increasingly well-explored in DCD grafts, while the impact on outcomes with the transplant of other ECD liver types requires further studies. This last subsection will highlight the current concepts of MP, with a focus on the clinical results after transplantation, considering the initial donor/recipient risk and the underlying mechanism of protection or injury.

### 5.1. Concepts of Normothermic Organ Perfusion

Human beings naturally expect the superiority of normothermic perfusion techniques over every other strategy performed in the cold due to the “healthy” appearance of organs during a perfusion at 37 °C with a blood-based perfusate [113]. Organs appear red, hearts may beat and livers produce bile. Despite the increasing experience with organ assessments, the occurring IRI-associated inflammation does not immediately affect the macroscopic appearance and may therefore lead to the impression that an organ is “healthy”. Furthermore, the IRI level is not directly quantifiable, and many clinicians believe an organ requires the normothermic conditions to be assessed for its current metabolism or function, thereby sometimes neglecting that the reintroduction of oxygen causes the full picture of IRI when done under warm conditions [114].

**Figure 3:**
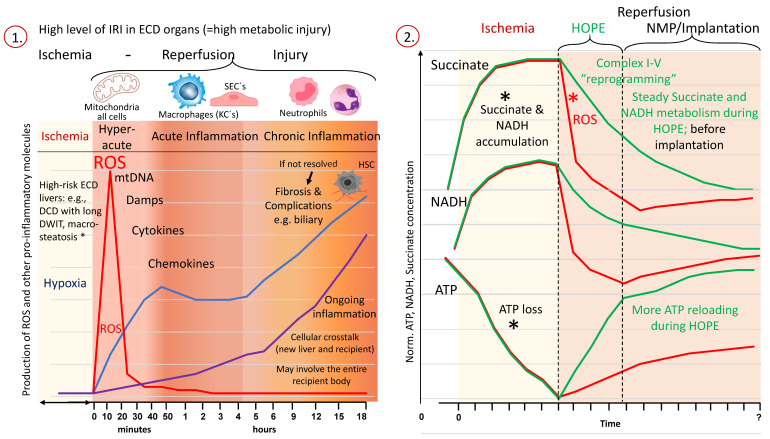

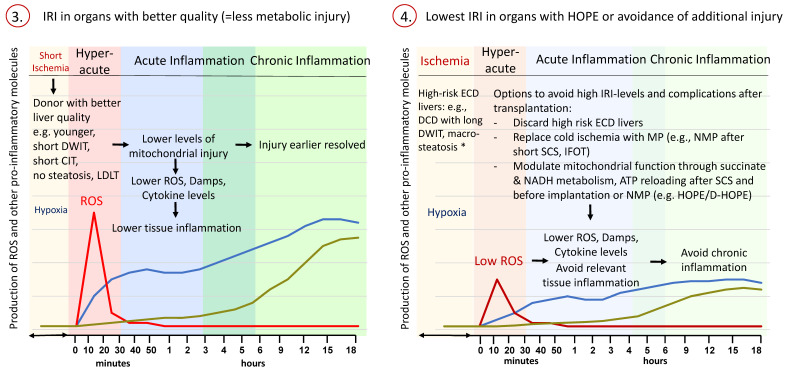
Cascade of ischemia–reperfusion injury (IRI) based on the liver quality. The metabolic features of IRI with different organ qualities are described in 1–4. Livers from ECD donors convey an elevated risk, sometimes even with short warm and cold ischemia, due to a low donor quality and prolonged ICU treatment. During ischemia, NADH and succinate accumulate to high levels with ATP loss at the same time. When oxygen is reintroduced, the injury becomes immediately visible with ROS, DAMPs and cytokine release throughout the 3 phases: first, the hyperacute and acute responses, which either resolve with recurrent liver function or transform into a chronic phase with ongoing inflammation, severe complications and graft loss; ROS release over time (red line); acute inflammation with Damps, cytokine and chemokine release over time (blue line); chronic inflammation (purple line) (**1**). When oxygen is reintroduced under warm conditions, the succinate and NADH are immediately and rapidly metabolized to reestablish an electron flow to rebuild ATP, which is urgently needed for all the cell functions, which return to their normal speed with high demands of ATP. This results in the severe proinflammatory status of some ECD livers after reperfusion. This injury is significantly reduced with hypothermic reoxygenation (HOPE and D-HOPE), where succinate and NADH are slowly metabolized with the recovery of the respiratory chain and ATP content without the immediate high demand of energy and full cellular function. Once the mitochondria have recovered, the implantation or normothermic reperfusion is less detrimental with less ROS, DAMPs and cytokines; green line (HOPE) and red line (unperfused controls livers with standard cold storage) demonstrate differences in Succinate, NADH and ATP levels from donation to postreperfusion after transplantation (**2**). The mechanisms were described in Reference [110]. Template 3 and 4 demonstrate the ischemia-reperfusion injury cascade compared to template 1 with different risk profiles. When ischemia is shorter or the graft of better quality, the acute and chronic injury are lower (**3**). The 4th panel shows the protective strategies currently applied to reduce the IRI-associated consequences. (**4**). When HOPE is performed before implantation or the injury reduced to low levels or cold storage replaced by perfusion throughout, the level of inflammation after reperfusion is very limited and shown with low ROS, Damps and cytokine levels, related to those in template 1. DBD: donation after brain death; DCD: donation after circulatory death; ICU: intensive care unit; HOPE: hypothermic oxygenated perfusion; Panel 2: Green line: HOPE and normothermic reperfusion; Red Line: direct normothermic reperfusion. * Rapid Succinate metabolism → ROS and Complex I and II dysfunction, * in Panels 1 and 4: high-risk ECD organs (e.g., extended DCD and macrosteatosis) accumulate enormous levels of NADH and succinate and more loose energy. ATP: adenosine trisphosphate; ECD: extended criteria donors; HOPE: hypothermic oxygenated perfusion; IFOT: ischemia-free organ transplantation; ROS: reactive oxygen species; SCS: standard cold storage. Figure done supported by biorender.com (assessed on 21 July 2022).

This is in clear contrast to the metabolism seen in mitochondria with very minimal ROS production and injury when tissues receive oxygen under hypothermic temperatures below the Arrhenius break point of 15 °C (Figure 3) [72,110,114].

Two main normothermic perfusion concepts are currently applied to human organs before implantation. First, normothermic regional perfusion (NRP), where the donor blood is reoxygenated and recirculated after canulation, and when the donor WIT and stand-off periods have passed [115]. This approach, routinely performed for 1–4 h, prolongs the required theater time with the donor compared to super rapid retrieval (SRR) and cold storage and requires additional labor support (e.g., circulating nurse) and a transfer of perfusion equipment to the donor site [116,117].

The leading countries using this technique routinely for DCD liver procurement are Spain, France, Italy and the United Kingdom (UK) [41,118,119]. During NRP, the abdominal organs release the well-known proinflammatory molecules of IRI, and some are used to assess and predict the liver performance after later implantation [112,117]. In addition to liver macroscopy and biopsy, perfusate lactate and transaminases are routinely measured with various guidelines in different countries [116,120].

The results after transplantation are available from multiple local or national case–control studies demonstrating the superiority of NRP combined with cold storage compared to the standard cold storage alone. Based on the opportunity for donor cannulation prior to treatment withdrawal in Spain, the duration of functional DWIT appears rather short, with 10–14.4 min in the recent series (Table 2).

A large Spanish series of 545 controlled DCD livers procured with NRP showed excellent outcomes, provided the cold storage after NRP and the recipient risk was controlled [119]. Such results paralleled earlier findings from France, where the breaching of the current guidelines led to more graft loss due to PNF and liver cancer recurrence [121]. Candidates with a lab MELD of <25 points and no additional technical challenges (e.g., higher grade portal vein thrombosis) who received a DCD liver of up to 65 y of age with <30 min fDWIT procured with NRP and maximal 8-h cold storage achieve equally good results, as seen with standard DBD liver transplants [121,122].

Of great interest is also the recent comparative study from Cambridge. Watson et al. analyzed the outcomes after DCD LT comparing NRP with normothermic machine perfusion (NMP) [123]. In contrast to the two randomized controlled trials (RCT), where NMP was applied after a short cold storage of 2.1 and 2.9 h, in this retrospective, matched DCD cohort study, NMP is used after a median of 6.6 h cold storage at the recipient center [123,124,125]. Interestingly, the authors demonstrate almost the same rate of ischemic cholangiopathy in the NMP group compared to cold storage alone (19% vs. 25%) [123] and parallel earlier results from Birmingham published in 2020 (Table 3) [126].

In contrast, an early reoxygenation with NRP after DWIT and before 6.7 h of cold storage achieved better outcomes with less biliary complications and better graft survivals [123]. Of interest is also the functional DWIT in this comparative study (median 15–19 min), which appears similar as recent data from France (22 min), where an overall DCD donor recipient risk within national guidelines achieved excellent results after transplantation [121,122].

Based on the current literature, normothermic reperfusion seems more efficient when done instead or before cold storage, e.g., in the donor, with additional advantages conveyed by “natural” liver positioning and the contribution from other abdominal organs. Such results are further supported by a recent matched cohort study where NMP after short cold storage achieved similar results as DCD transplantation with NRP (Table 3) [123,126,127]. The role of normothermic perfusion techniques in other types of ECD organs, e.g., older donors or livers with advanced steatosis, is less well=explored. Livers with advanced steatosis experience relevant IRI levels after implantation or during NMP. Researchers are currently exploring the role of regenerative interventions during NMP, such as, for example, the concept of defatting, which may improve steatotic grafts, which overcome the initial IRI hit during prolonged NMP [113,128,129].

### 5.2. Hypothermic Perfusion Organ Perfusion

One remaining challenge to compare and identify the most beneficial MP techniques in the “jungle of literature” is inconsistent risk reporting. DCD livers with extended donor risks are routinely transplanted in Italy, where a 20-min stand-off period contributes to a prolonged functional DWIT of 40–43 min [41,42]. Most Italian centers combine NRP with ex situ MP, mainly hypothermic techniques, and achieve excellent results with very low IC-rates and no liver-related graft loss (Table 4) [35,41,42,130].

Hypothermic perfusion techniques are routinely applied after standard procurement and transport in the recipient center. While, in the first clinical study, reported in 2010, an artificial perfusion solution without additional oxygenation (pO_2_: 20 kPa) was used, the importance of a high-perfusate oxygen concentration has become increasingly understood [131]. When oxygen becomes reintroduced into ischemic tissues under cold conditions (e.g., below 15 °C), the respiratory chain in most organs can recover, despite high levels of previous injury and, importantly, with a much lower ROS release compared to normothermic conditions [72,110,114,132].

The reason behind this is the slow restart of aerobe oxygenation with a forward electron flow, the subsequent slow and steady metabolism of accumulated NADH and succinate and effective ATP-reloading. These mechanisms were demonstrated in various solid organs, not only in ECD livers [110,132,133,134,135,136]. With such mitochondrial priming, the later warm reperfusion at transplantation or during NMP is less detrimental with lower ROS, DAMPs and cytokine release [110,114,132]. The clinical correlate is increasingly explored in many studies, including three published RCTs with DCD livers and old and steatotic grafts [72,137,138,139,140,141]. The use of HOPE and D-HOPE after the standard cold storage was found to significantly reduce the transaminase release and related EAD rates, to shorten the ICU and hospital stay and, most importantly, to reduce the number and severity of ischemic cholangiopathy, seen in ECD grafts from old donors and in DCD liver transplants (Table 5) [137,138,141,142].

**Figure 4:**
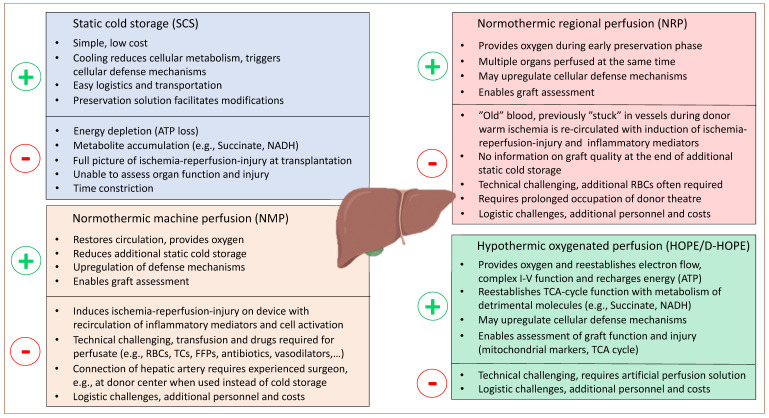
Advantages and disadvantages of different types of liver preservation. ATP: adenosine trisphosphate; DBD: donation after brain death; DCD: donation after circulatory death; FFP: fresh frozen plasma; hope: hypothermic oxygenated perfusion; ICU: intensive care unit; NADH: nicotinamide adenine dinucleotide hydrogen; NMP: normothermic machine perfusion; NRP: normothermic regional perfusion; RBC: red blood cell concentrates; SCS: standard cold storage; TCA: tricarboxylic acid (cycle). Figure done supported by biorender.com (assessed on 21 July 2022).

In addition to NMP, hypothermic concepts are also increasingly used to prolong the overall preservation with prospective ongoing studies to safely move transplantation into the daytime [143,144,145,146]. With a prolonged cold ischemia time, split grafts are another source of ECD livers, which will benefit from the early reintroduction of oxygen in the cold to reduce inflammation. The first case series were reported from different countries using all types of splits, performed during or before HOPE treatment [103,147,148,149,150]. The results from ongoing studies are awaited and may increase the use of this technology in other countries with limited deceased donors to optimize the number of available organs for the pediatric population (Figure 4).

To identify the best combination of viability markers, which enable the reliable outcome prediction during MP is another key target today. To achieve this high-end goal, the underlying mechanisms of IRI and their instigators should be considered. Biochemical molecules are quantified in perfusates and bile during all sorts of MP with controversial results, however, and modifications of the suggested parameters with threshold shifts, presented by the same groups, are ongoing [21,151,152]. There is a clear need for a larger case series and validation studies using a standardized perfusion technique to enable the comparison of the transplant risk in correlation with the outcomes. To ultimately proove if rewarming and NMP are required to predict biliary complications after LT, the role of mitochondrial markers should be explored further with different perfusion techniques [110,153].

**Table 2:** Clinical studies with normothermic regional perfusion (NRP) and ECD grafts (DCD Type III) within the last 3 years.

Study Type	Reference	Number and Type of Livers	Criteria to Define ECD Livers	Type and Duration of DWIT (Min)	NRP-Duration (Min)	CIT after NRP (h)	Discard Rate	Follow-Up (Months)	Main Findings	Discussion
Case-control cohort study, retrospective (Level of Evidence IV)	Schurink et al. [120]	25 DCD NRP vs. 49 DCD SCS vs. 81 DBD SCS	ET-DRI: 3.1 (2.97–3.21) vs. 2.19 (1.9–2.42) vs. 1.69 (1.49–2.01), UK-DCD Risk: 9 (8–12) vs. 6 (5–9)	NRP vs. SCS: fDWIT 29 (range 26–33) vs. 24 (19–28)	120 (range 110–128)	5.7 (range 4.9–6.45)	5 discarded after NRP	NRP: 23 (range: 14–28)	DCD NRP vs. DCD SCS vs. DBD: additional D-HOPE in 5 (25%) vs. 19 (39%) vs. 3 (4%). 1-year graft survival: 90% vs. 82% vs. 86%, LoHS 13 (10–18) vs. 14 (11–20) vs. 17 (12–27); IC 11% vs. 18% vs. 7%	Low case number, heterogenous cohort, pilot
	Rodriguez et al. [154]	39 DCD NRP vs. 78 DBD SCS	-	fDWIT 13.1 (6–26), tDWIT 19.23 (10–38)	90–120	NRP DCD vs. DBD 5.0 vs. 5.2	-	mean 22	NRP vs. DBD: PNF 0% vs. 3.8%, EAD 34.2% vs. 19.2%, biliary complications early 5.1% vs. 3.8%, late 7.6% vs. 12%, IC 0% versus 1.2%, LoHS 14.7 (1–51) vs. 13.7 (7–57)	No cold storage DCD control
	Hessheimer et al. [119]	545 DCD NRP vs. 258 DCD SCS	UK DCD risk: 27 (5%) vs. 15 (5.8%) futile	NRP vs. SCS: fDWIT 12 (9–16) vs. 14 (11–20); tDWIT (18 (13–23) vs. 22 (17–26)	111 (81–126)	NRP vs. SCS: 5.3 (4.5–6.3) vs. 5.6 (4.7–6.5)	-	median 31	NRP vs. SCS: PNF 16 (3%) vs. 15 (6%); HAT 22 (4%) vs. 19 (7%); biliary complications 63 (12%) vs. 75 (29%); ITBL 6 (1%) vs. 24 (9%), graft loss 77 (14%) vs. 88 (34%)	Large cohort, retrospective, short DWIT
	Ruiz et al. [155]	100 DCD NRP vs. 200 DBD SCS	UK DCD risk: 3 (3%) futile	fDWIT: 10 (IQR: 8.5–12.2)	121 (IQR: 118–128)	NRP vs. SCS: 4.6 (4–5.2) vs. 4.4 (3.8–5.7)	-	Median 36 (20–48.3)	similar results, same EAD rate and enzyme release, 3-year graft survival 92% vs. 87%	Retrospective matched, short cold storage, short DWIT
	Muñoz et al. [156]	23 DCD NRP vs. 22 DCD SCS	-	NRP vs. SCS: tDWIT: 23.7 vs. 23.1; fDWIT: 14.4 vs. 15.8	90–120	NRP vs. SCS: 4.7 vs. 4.7	-	NRP 14.4 vs. SCS 34.8	NRP vs. SCS: EAD rate 30.5% vs. 68.1%; overall biliary complications 4.3% vs. 22.7%; IC rates 0–5% vs. 13.6%, re-transplantation 0% vs. 9.1%	shorter follow up in NRP group: severe complications may develop later, small case load, short DWIT
	Savier et al. [122]	50 DCD NRP vs. 100 DBD SCS	National DCD guidelines *	fDWIT: 22 (IQR: 20–26.8); Asystolic DWIT: 17 (IQR: 14–22.3);	Median 190 (IQR: 151–223)	DCD NRP: 5.8 (5–6.7), DBD SCS: 6.3 (5.4–7.3)	-	≥24	DCD NRP vs. DBD SCS: EAD 18% vs. 32%; AKI 26% vs. 33%; 12% graft loss within 2 years (HCC recurrence) vs. 3%; overall biliary complications 16% vs. 17%	Retrospective matched study, transparent presentation of utilization
	Hessheimer et al. [115]	95 DCD NRP vs. 117 DCD SCS	-	NRP vs. SCS: tDWIT: median 20 vs. 21, fDWIT: median 14 vs. 13	120 (range 79–136)	NRP vs. SCS: 5.3 (4.4–6.1) vs. 5.7 (4.8–6.4)	-	≥12 (median 20)	NRP vs. SCS: EAD 22% vs. 27%, same rate of PNF and HAT, overall biliary complications 8% vs. 31%, IC 2% vs. 13%, graft loss 12% vs. 24%	Retrospective, large cumulative cohort
	Watson et al. [157]	43 DCD NRP vs. 187 DCD SCS	DRI: 1.8 (1.7–2.4) vs. 2.5 (2–2.9)	NRP vs. SCS: tDWIT: 30 (26–36) vs. 27 (22–32); asystolic DWIT: 16 (13–20) vs. 13 (11–16)	Median 123 (IQR: 103–130)	6.4 (5–8.4) vs. 7.4 (6.6–8.2)	-	≥3	NRP vs. SCS: 0% graft loss due to IC vs. 6%; similar graft survival but shorter follow up in NRP group	Despite shorter follow-up (NRP), survival comparisons calculated

Extended criteria donor livers transplanted with NRP and cold storage within the last 3 years. Cohorts without a control group were excluded, and only studies with a minimal case number of 20 grafts were considered. AKI: acute kidney injury; CIT: cold ischemia time; DBD: donation after brain death; DCD: donation after circulatory death; DRI: donor risk index; DWIT: donor warm ischemia time; fDWIT: functional DWIT; tDWIT: total DWIT; EAD: early allograft dysfunction; ET DRI: Eurotransplant DRI; HAT: hepatic artery thrombosis; IC: ischemic cholangiopathy; ITBL: ischemic-type biliary lesion; ICU: intensive care unit; LoHS: length of hospital stay; MP: machine perfusion; NMP: normothermic machine perfusion; NRP: normothermic regional perfusion; PNF: primary nonfunction; SCS: standard cold storage; UK: United Kingdom. * Donor age ≤ 65 y, fDWIT ≤ 30 min, SCS ≤ 8 h, <20% macrosteatosis; recipient age ≤ 65 y, lab MELD ≤ 25, no re-transplantation or previous surgery; AST/ALT < 200 during NRP.

**Table 3:** Clinical studies with normothermic machine perfusion (NMP) and ECD (DCD Type III) grafts within the last 3 years.

Study Type (Level)	Reference	Number and Type of Livers	Criteria to Define ECD Livers	Type and Duration of DWIT (Min)	CIT before NMP (h)	CIT in Control	NMP-Duration (h)	Discard Rate	Follow-Up (Months)	Main Findings	Discussion
RCT (II)	Markmann et al. [125]	DBD/ECD/DCD livers: 151 NMP vs. 142 SCS	Age > 40 y, CIT > 6 h, DCD (inclusion: donor age < 55 y, macrosteatosis < 40%)	not available	2.9 ± 1.53	5.6 ± 1.5	4.6 ± 1.96	Discarded: 2 NMP, 5 SCS	6	NMP: histologically less IRI (*p* = 0.004), reduction of IC (*p* = 0.02), EAD (*p* = 0.01)	heterogenous population: DBD, ECD, DCD, no report of DWIT, focus on EAD as endpoint (not powered for)
	Nasralla et al. [124]	55 DCD NMP vs. 34 DCD SCS; 87 DBD NMP vs. 80 DBD SCS	55/34 DCD livers, cold storage; ET DRI: 1.7 (1.47–2.07) vs. 1.71 (1.5–2.01)	fDWIT NMP: 21 (IQR: 17–25), SCS: 16 (IQR: 10–20)	2.1 (1.8–2.4)	7.8 (6.3–9.6)	9.1 (6.2–11.8) DBD: 9.9; DCD 8.8	64/334 excluded (19.1%), 48/270 discarded (17.8%)	12	Lower liver enzyme release after NMP (primary endpoint), no differences in biliary complications or graft survival	High exclusion/discard rate; control group with higher injury, no report on perfusate transaminases, NMP replacing SCS
Prospective, matched case control (III)	Fodor et al. [158]	59 DBD/DCD with NMP; matched with 59 SCS	ET DRI: 1.78 (0.51) vs. 1.85 (0.72)	not available	6	7	* estimated duration of NMP: 15 (total preservation time: 21)	16/75 (20%) discarded after NMP	≥3	NMP vs. SCS: patient and graft survival 81% vs. 82%. Same rate of major complications, lower biliary complications with NMP (*p* = 0.047)	short follow-up, retrospective, high rate of biliary complications in DBD SCS control group
	Mergental et al. [126]	10 DCD NMP vs. 12 DBD NMP vs. 44 SCS (matched controls)	Overall US DRI: 2.1 (1.9–3)	fDWIT 22.5 (IQR:19.0–35.0)	DCD 6.9 (5.9–7.7); DBD 8.5 (6.8–12)	not available	9.8 (7.5–11.8)	9/31 (29%) not transplanted; 3/25 (12%) discarded after NMP	12	DCD/DBD vs. SCS: EAD: 7 (31.8%) vs. 4 (9.1%); NAS: 4 (18.2%, 3/4 DCDs) vs. 1 (2.3%) (4/22; 3 DCD); 80% of DCD recipients had PRS	Prospective study with retrospective matched control, heterogenous risk profile
Case-control cohort study, retrospective (IV)	Ceresa et al. [159]	23 DBD/8 DCD with SCS-NMP vs. 104 only NMP from [125]	DRI 1.87 (range 1.06–3.2) vs. 1.45 (0.78–6.35)	SCS-NMP vs. only NMP: fDWIT 16 (range: 12–28) vs. 20 (range:10–35)	6 ± 1.3	No nonperfused control	SCS-NMP 8.4 ± 4 vs. only NMP 12 ± 4.2	20/51 (39%) excluded	12	Comparable outcomes among SCS-NMP vs. only NMP (EAD, PRS, hospital/ITU stay); similar rate of major complications; 1 y graft survival: 84% vs. 94%;	Standard risk DBD and DCD grafts, 8 DCD only
	Quintini et al. [160]	21 discarded DCD/DBD with NMP: 6 discarded; 15 transplanted	Macrosteatosis >30%, combined up to 60%; hypertransaminase	fDWIT 21 (±10)	5.4 ± 1.1; DBD: 4.8 ± 1.4; DCD: 5.5 ± 1.2	No control	6.7 ± 2.1; DBD: 7.1 ± 0.9; DCD: 7.7 ± 2.9	6/21 (29%) discarded	2–14	Hospital stay: DBD 9.5 ± 4.4 vs. DCD 19.5 ± 10.2; 1 × IC, 7/15 EAD;	

Extended criteria donor livers transplanted with NMP and cold storage within the last 3 years. Cohorts without a control group were excluded, and only studies with a minimal case number of 20 grafts were considered. CIT: cold ischemia time; DBD: donation after brain death; DCD: donation after circulatory death; DRI: donor risk index (US: Feng S et al. [60]); DWIT: donor warm ischemia time; fDWIT: functional DWIT; tDWIT: total DWIT; EAD: early allograft dysfunction; ET-DRI: Eurotransplant DRI; IC: ischemic cholangiopathy; ICU: Intensive care unit; MP: machine perfusion; NMP: normothermic machine perfusion; NRP: normothermic regional perfusion; PNF: primary nonfunction; PRS: postreperfusion syndrom; RCT: randomized controlled trial; SCS: standard cold storage; UK: United Kingdom. * Calculated by the review’s authors.

**Table 4:** Case–control studies with combined preservation techniques and ECD grafts (DCD Type III) within the last 3 years.

Protocol.	Reference	Number and Type of Livers	Criteria to Define ECD Livers	Type and Duration of DWIT (min)	CIT after NRP/before MP (h)	Perfusion (h)	Discard Rate	Follow-Up (Months)	Main Findings	Discussion
NRP + SCS vs. SCS + NMP vs. SCS alone	Gaurav et al. [123]	69 DCD NRP vs. 67 DCD NMP vs. 97 DCD SCS	DRI: 2.2 (1.8–2.5) vs. 2.5 (2–2.9) vs. 2.5 (2–3), UK DCD Risk: 9 vs. 3 vs. 1 futile	NRP vs. NMP vs. SCS: fDWIT 19 (15–24), 15 (12–18), 15 (11–18); tDWIT: 29 (23–33), 26 (22–31), 26 (22–31)	NRP vs. NMP vs. SCS: 6.7 (5.7–7.9) vs. 6.6 (5.8–7.4) vs. 7.2 (6.6–7.9)	NRP vs. NMP: 2.2 (2–2.4) vs. 7.7 (5.5–9.5)	-	median 38	NRP vs. NMP vs. SCS: PNF 0% vs. 1.5% vs. 5%, EAD 14% vs. 11% vs. 21%. Total biliary complication: 22% vs. 37% vs. 42%; IC: 6% vs. 19% vs. 25%; HAT 1% vs. 8% vs. 8%. LoHS 15 (13–23) vs. 19 (13–29) vs. 18 (15–30)	Large cohort, NRP vs. NMP vs. SCS, relevant clinical endpoint, not randomized
NRP + SCS vs. SCS + NMP	Mohkam et al. [127]	157 DCD NRP vs. 34 DCD NMP	DRI: 1.98 (1.68–2.43) vs. 2.13 (1.9–2.42)	NRP vs. NMP: tDWI: 31 vs. 25; asystolic DWIT 18 vs. 12	NRP: 5.8 NMP: 2.3	NRP: 3.1, NMP: 8.8	-	23	NRP vs. NMP: 1 PNF, 1 hep vein thrombosis, hyperacute rejection, 2 HAT in NRP, 1 Cava thrombosis, HAT, graft infraction in NMP; 11.8% vs. 20.6% biliary complication (ns), anastomotic strictures 8.8 vs17.6%, NAS 1.5 vs2.9%; LoHS 14 (8–17) vs. 16 (13–20)	no control, short cold storage prior to NMP
NRP + HOPE/D-HOPE vs. SCS alone	Patrono et al. [42]	20 DCD with NRP + D-HOPE vs. 40 DBD SCS	-	fDWIT 43 (IQR 35–46)	DCD vs. DBD 4.4 (3.8–4.9 vs. 7 (6.3–8.5)	NRP 4.1 (3.7–4.5), D-HOPE 3.4 (2.4–4.6)	none	DCD 15.5 m (12–27), DBD 40 (21–56)	DCD vs. DBD: EAD 1% vs. 28%; patient-survival 100% vs. 95%, graft-survival 90% vs. 95%. Biliary strictures anastomotic 22% vs. 15%, NAS 18% vs. 10%	Single centre, retrospective, matched, high donor risk (DWIT)
	De Carlis et al. [41]	37 DCD with NRP + D-HOPE matched with 37 SCS	UK DCD risk: 24 (Italy) vs. 4 (UK) futile	NRP/D-HOPE vs. NRP/SCS: fDWIT 40 (IQR: 30–80) vs. 18 (IQR: 10–44)	NRP/D-HOPE: 6.9 (5.5–11); NRP/SCS: 6.5 (4–9.7)	NRP: 4.2 (0.9–7.7); D-HOPE: 2 (0.7–6.3)	86.6% transplanted after NRP	≥12	Despite longer DWIT DCD with NRP + D-HOPE showed less biliary complications and better graft survival as DCD with SCS alone	High donor risk, prolonged DWIT, shorter follow up in perfusion group; retrospective
NRP + HOPE vs. NRP + SCS	Maroni et al. [161]	36 DCD total; 19 NRP + HOPE vs. 17 NRP + SCS	UK DCD risk: 11 (range 6–16) vs. 7 (range: 3–12)	NRP + HOPE vs. NRP + SCS: tDWIT 56.65 ± 20.4 vs. 39.1 ± 21.6; fDWIT: 41.9 ± 12.5 vs. 25.5 ± 3.7; asystolic DWIT: 30.5 ± 7.7 vs. 20.5 ± 4.1	NRP + HOPE: 7.9 ± 1.4, NRP + SCS: 6.5 ± 2.9	NRP + HOPE: NRP: 3.8 ± 1.1, HOPE: 2.5 ± 1.1; NRP + SCS: NRP: 3.3 ± 0.8	-	Median 24	Italian DCD with NRP + HOPE had 0% IC compared to NRP/SCS (France) 12.5%; additional HOPE after NRP plus SCS improves outcomes	Higher risk in Italy, retrospective, matched cohorts
NRP + HOPE vs. SCS + HOPE	Dondossola er al. [162]	28 DBD (ECD) DHOPE/HOPE vs. 22 DCD: NRP + DHOPE/HOPE	ECD definition: Vodkin et al. [163]	tDWIT 54 (IQR: 40–66) fDWIT 40 (33.5–51)	DBD (ECD) vs. DCD: 9.7 (7.8–11.1) vs. 8.3 (7.0–9.4)	NRP: 4 (3–5) DBD (ECD) vs. DCD: HOPE: 2.7 (1.6–3.5) vs. 3 (1.5–4)	-	17 (IQR: 10–26)	1 PNF (DCD), 5 EAD each group, 3 biliary complications (1 leak, 2 stenosis) (DBD), 1 ITBL (DCD), CIT > 9 h with prolonged hospital stay, higher rates of EAD, worse complications	Two centers, two DHOPE and HOPE, 7% have 6 months FU
NRP + SCS vs. SCS + HOPE	Muller et al. [153]	DCD: 132 NRP vs. 93 HOPE	US DRI: 2.01 (1.75–2.31) vs. 2.47 (2.08–2.8); UK DCD risk: 12 (9.1%) vs. 42 (45.2%) futile	tDWIT 31 (26–36) vs. 35 (30–39); fDWIT 22 (19–26) vs. 31 (26–35), *p* < 0.001	5.7 (4.7–6.6) vs. 4 (3.1–5)	NRP: 3.1 (2.7–3.5); HOPE: 2.2 (1.8–2.8)	-	20 (9–25) vs. 28 (15–248)	NRP vs. HOPE: No differences in LoHS, PNF, IC, art. complications. biliary complication 23 (17.4%) vs. 32 (34.4%), *p* = 0.004; AS 14 (10.6%) vs. 24 (25.8%), *p* = 0.003	More donor risk in HOPE cohort, retrospective matched
SCS + COR	Van Leeuwen et al. [152]	DCD: 54 COR: 24 transplanted (12 HBOC vs. 22 RBC)	ET-DRI: 2.91 (2.6–3.16) vs. 3.12 (2.63–3.38)	Transplanted vs. discarded: fDWIT 29 (25–35) vs. 32 (26–35)	4.5 (4.1–4.9) vs. 4.8 (4.5–5.8)	1 D-HOPE, 1 COR, ≥2.5 NMP	20 discarded	HBOC: 38 (34–41); RBC: 17 (13–25)	HBOC vs. RBC: similar results for patient and graft survival and complications	Heterogenous cohort with different perfusates, retrospective
	Van Leeuwen et al. [164]	DCD: 11 COR vs. 36 DBD/SCS vs. 24 DCD/SCS	ET-DRI: 2.81 (2.6–2.9) vs. 1.75 (1.48–1.9) vs. 2.34 (2.14–2.49)	COR vs. DCD: tDWIT: 32 (25–33) vs. 28 (23–33); asystolic DWIT: 16 (14–16); 16 (14–20)	DCD/COR: 4.6 (4–4.9); DCD/SCS: 7.4 (6.3–8.2); DBD/SCS: 6.8 (5.9–7.9)	1 D-HOPE + 1 COR + ≥ 2.5 NMP total NMP 6.7–9)	-	Median 12 m (8–22 m)	9% IC after COR compared to 18% in DCD/SCS control group, higher rate of AS after COR (27% vs. 18% in DCD/SCS control	Retrospective, unperfused DCD with higher risk, longer SCS in control group

Extended criteria donor livers transplanted with combined perfusion approaches within the last 3 years. Cohorts without a control group were excluded, and only studies with a minimal case number of 20 grafts were considered. AS: anastomotic stricture; COR: controlled oxygenated rewarming; CIT: cold ischemia time; DBD: donation after brain death; DCD: donation after circulatory death; DRI: donor risk index; DWIT: donor warm ischemia time; EAD: early allograft dysfunction; ET-DRI: Eurotransplant DRI; HBOC: hemoglobin-based oxygen carrier; ICU: intensive care unit; LoHS: length of hospital stay; MP: machine perfusion; NMP: normothermic machine perfusion; NRP: normothermic regional perfusion; PNF: primary nonfunction; RBC: red blood cells; SCS: standard cold storage; UK: United Kingdom.

**Table 5:** Clinical studies with hypothermic oxygenated perfusion (HOPE and D-HOPE) and ECD grafts within the last 3 years.

Study Type (Level)	Reference	Number and Type of Livers	Criteria to Define ECD Livers	Type and Duration of DWIT (min)	CIT before HOPE/D-HOPE (h)	CIT in Control (h)	Duration of HOPE/D-HOPE (h)	Discard Rate	Follow-Up (Months)	Main Findings	Discussion
RCT (II)	Ravaioli et al. [139]	55 DBD (ECD) per arm (HOPE vs. SCS)	UNOS criteria for ECD; US DRI 1.85 (1.72–1.9) vs. 1.77 (1.55–1.9)	No DCD livers	4.3 (3.6–5.4)	7 (6–7.5)	2.4 (2–3.1)	54/55 and 52/55 achieved primary endpoint	15.7	HOPE vs. SCS: EAD: 13% vs. 35%, *p* = 0.007; Re-TPL: 0% vs. 11%, *p* = 0.03; biliary/vascular complictions similar, HOPE with lower rate of acute/chronic rejection and cardiovascular events; graft failure higher in SCS (*p* = 0.03)	Power analysis done for combined study with livers and kidneys, endpoint EAD
	Van Rijn et al. [137]	78 DCD per arm (D-HOPE vs. SCS)	US DRI: 2.12 (1.84–2.38) vs. 2.12 (1.86–2.42)	D-HOPE vs. SCS: tDWIT 29 (IQR: 22–33) vs. 27 (IQR: 21–35); Asystolic DWIT: 11 (IQR: 8–13) vs. 11 (IQR: 8–15)	6.2 (5.9–6.9)	6.8 (5.9–8)	2.2 (2–2.5)	156/160 achieved primary endpoint	6	D-HOPE significantly reduces IC rates (*p* = 0.03) and the number of required interventions, less EAD, less acute rejections	Follow-up only 6 months
	Czigany et al. [138]	23 DBD (ECD) per arm (HOPE vs. SCS)	German medical chamber *; ET-DRI: 2.05 (1.88–2.2)	No DCD livers	6.3 (5.2–7.8)	8.4 (7.8–9.7)	2.4 (1.7–3.4)	no drop out	12	HOPE treatment reduced peak ALT levels (*p* = 0.03), ICU (*p* = 0.045)/hospital stay (*p* = 0.002), major complications (*p* = 0.036), CCI (*p* = 0.021), costs (*p* = 0.016)	Study was not powered for complications
Case-control cohort study, retrospective (IV)	Patrono et al. [141]	DBD (ECD): 121 D-HOPE vs. 723 SCS	-	No DCD livers	5.8 (5.3–6.65)	7.3 (6.5–8.2)	2.3 (1.9–3)	-	D-HOPE 22, SCS 47.3	D-HOPE with EAD reduction (*p* = 0.024), CCI (*p* = 0.003), lower IC severity in D-HOPE (*p* = 0.007). Subgroup with elderly donors: same results	Retrospective mached, ECD-DBD grafts
	Rayar et al. [165]	DBD (ECD), DCD: 25 HOPE vs. 69 SCS	Age > 65 y, BMI > 30 kg/m^2^, ICU stay > 7 d, Na^+^ > 155 mmol/L, ALT/AST > 3 x normal, macrosteatosis >30%	not available	8.8 (range: 6.3–13.7)	9.3 (range: 3.5–12)	1.95 (range: 1.3–4.2)	-	12	HOPE with lower recipient ALT, shorter ICU/ hospital stays, HOPE vs. SCS: AS 8% (*n* = 2/25) vs. 10.1% (7/69); Leaks 0% (*n* = 0/25) vs. 1.4% (1/69; ischemic), no cost difference	Retrospective, matched, DBD and DCD livers mixed
	Schlegel et al. [142]	50 DCD HOPE vs. 50 DCD SCS vs. 50 DBD SCS	UK DCD risk score	DCD HOPE vs. DCD SCS: tDWIT: 36 (IQR: 31–40) vs. 25.5 (IQR: 21–31); fDWIT: 31 (IQR: 27–36) vs. 17 (IQR: 15–19); Asystolic DWIT: 19 (IQR: 17–21) vs. 12.5 (10–15)	4.4 (3.7–5.2)	DCD-SCS: 4.7 (4.3–5.3) DBD-SCS: 5 (4–5)	2 (1.6–2.4)	-	60	DCD HOPE vs. DCD SCS vs. DBD SCS: AS 24% (*n* = 12/50) vs. 18% (*n* = 9/50) vs8% (4); 1 biliary leak each group (2%), IC 8% (*n* = 4/50) with 0% graft loss vs. 22% (*n* = 11/50) with 10% (*n* = 1/69) graft loss; DCD HOPE with less PNF, HAT and IC	Retrospective matched cohort study
	Patrono et al. [166]	DBD (ECD): 25 HOPE vs. 50 SCS	Age > 80 y, BMI > 30 kg/m^2^, CIT > 10 h; DRI 2.09 (0.52) vs. 2.15 (0.42)	No DCD livers	5.2 ± 0.9	6.5 ± 1.2	3.1 ± 0.8	-	6	HOPE: lower rate of PRS, AKI grade 2–3 and EAD. HOPE vs. SCS: biliary complications 16% vs. 12%	Retrospective atched cohort study, ECD-DBD grafts
	Rossignol et al. [103]	40 split liver TPL: 8 HOPE splits vs. 12 SCS	Standard split criteria in France	No DCD livers	Adults: 7.2 (6.6–8.5); Pediatric 8.2 (7.8–8.6)	Adults: 8.9 (7.5–10); Pediatric: 9.1 (8.6–9.5)	Adult: 2.6 (2.1–2.8); Pediatric: 1.6 (1.4–2.1)	-	7.5	similar outcome with low complication rate, 1 graft/patient loss in the pediatric SCS-group	

Extended criteria donor livers transplanted with hypothermic perfusion approaches and cold storage within the last 3 years. Cohorts without a control group were excluded, and only studies with a minimal case number of 20 grafts were considered. AKI: acute kidney injury; AS: anastomotic stricture; CCI: comprehensive complication index; CIT: cold ischemia time; DBD: donation after brain death; DCD: donation after circulatory death; DRI: donor risk index; DWIT: donor warm ischemia time; fDWIT: functional DWIT; tDWIT: total DWIT; EAD: early allograft dysfunction; ET-DRI: Eurotransplant DRI; ICU: intensive care unit; MP: machine perfusion; NMP: normothermic machine perfusion; NRP: normothermic regional perfusion; PNF: primary nonfunction; PRS: postreperfusion syndrom; SCS: standard cold storage; TPL: transplantation; UK: United Kingdom; y: years. * Age ≥ 65, ICU ≥ 7 d, BMI > 30 kg/m^2^, macrosteatosis > 40% or mixed, Na+ > 165 mmol/L, AST/ALT > 3 xnormal, Bilirubin > 2 mg/dL.

## 6. Remaining Challenges and Future Perspectives

To establish improved preservation protocols of ECD organs with reliable assessment tools to understand the metabolic risk and to predict posttransplant outcomes is the holy grail of organ transplantation today. Despite the large body of literature presented in the last decade, the implementation of MP in routine practice appears slow, which is based on various challenges previously described. The heterogenous experience of transplant surgeons with donor risk profiles in different countries and centers contribute to the difficulties for the routine adoption of new MP techniques worldwide. Based on the lack of cost analyses, only a few techniques have already been commissioned, and further progress depends on convincing data obtained from regional cost–benefit analyses. Another hurdle appears with the frequent need to locally confirm excellent outcomes shown in another region or center with one specific perfusion technique. Additional work is required to establish more uniform guidelines for the donor risk, utilization rates, trial endpoints and perfusion techniques to enable the standardized use of MP for ECD organs and the comparison of risk factors and related outcomes [167]. Despite an increasing number of consensus meetings, which summarize expert suggestions, as seen, for example, in DCD LTs, the implementation of guidelines in routine practice is time-consuming and requires persistence by already busy surgeons and other physicians involved. Similar facts apply with the viability assessment criteria and definitions for liver utilization rates. Despite the large number of smaller, retrospective case studies, there is an ongoing lack of RCTs and relevant endpoints. Despite the slow decrease of this phenomenon in DCD livers, this challenge remains active in other types of ECD livers, including steatotic grafts, where the additional lack of a reliable fat quantification method is another relevant boundary. Such challenges result in the resistance to use specific ECD livers with a subsequently low rate of studies on the outcomes, as seen, for example, in advanced macrosteatosis.

## Data Availability

Not applicable.
